# Research progress on the role of type I vesicular glutamate transporter (VGLUT1) in nervous system diseases

**DOI:** 10.1186/s13578-020-00393-4

**Published:** 2020-03-04

**Authors:** Xianchao Du, Jiashuo Li, Minghui Li, Xinxin Yang, Zhipeng Qi, Bin Xu, Wei Liu, Zhaofa Xu, Yu Deng

**Affiliations:** Department of Environmental Health, School of Public Health, China Medical University, Shenyang, 110122 Liaoning People’s Republic of China

**Keywords:** Glutamate, CNS, PNS, VGLUT1, Nervous system disease

## Abstract

Glutamate (Glu) is the predominant excitatory neurotransmitter in the central nervous system (CNS). Glutamatergic transmission is critical for controlling neuronal activity. In presynaptic neurons, Glu is stored in synaptic vesicles and released by stimulation. The homeostasis of glutamatergic system is maintained by a set of transporters in the membrane of synaptic vesicles. The family of vesicular Glu transporters in mammals is comprised of three highly homologous proteins: VGLUT1-3. Among them, VGLUT1 accounts for the largest proportion. However, most of the Glu is transported into the synaptic vesicles via the type 1 vesicle Glu transporter (VGLUT1). So, the expression of particular VGLUT1 is largely complementary with limited overlap and so far it is most specific markers for neurons that use Glu as neurotransmitter. Controlling the activity of VGLUT1 could potentially modulate the efficiency of excitatory neuro-transmission and change the filling level of synaptic vesicles. This review summarizes the recent knowledge concerning molecular and functional characteristic of VGLUT1, their development, contribution to a series of central nervous system and peripheral nervous system diseases such as learning and memory disorders, Alzheimer’s disease, Parkinson’s disease and sensitized nociception or pain pathology et al.

## Background

**Fig. 1:**
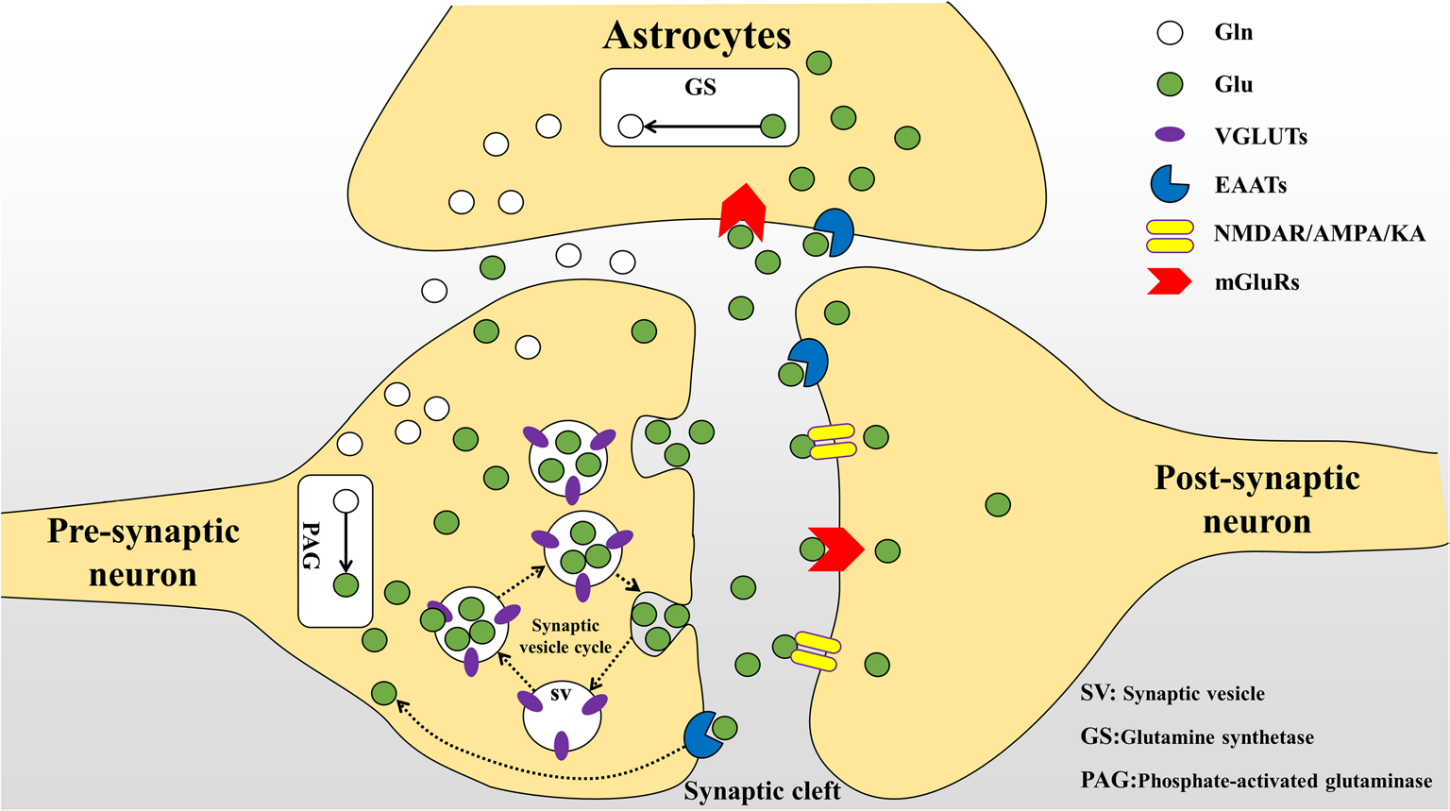
Glutamate metabolism cycle: After release of glutamate from the presynaptic neurons, a small percentage of glutamate is taken up by post-synaptic glutamate receptors and transporters. While the majority of this synaptically-released glutamate diffuses out of the synaptic cleft is taken up by astrocytic EAATs and mGluRs. In astrocytes, glutamate could be converted to glutamine by glutamine synthetase (GS), then glutamine is released to the synaptic cleft to be taken up by presynaptic neurons and used to resynthesize glutamate. The synaptic vesicles (SVs) uptake the resynthesize glutamate by VGLUTs to release back to the synaptic cleft through synaptic vesicles cycle

Glutamate (Glu) is a ubiquitous amino acid that is required by all cells for both protein synthesis and intermediate metabolism, It is also the major excitatory neurotransmitter in the central nervous system (CNS). Moreover, it plays an important role in memory, synaptic plasticity, neuronal development and neuronal activity [1–3]. After Glu releasing from pre-synaptic neurons, a small fraction of Glu is taken up by post-synaptic neuronal receptors. Meanwhile most of the Glu is released into the synaptic cleft and cleared by astrocytes. At the end, the remaining of Glu is up taken by pre-synaptic neurons themselves [4–7]. Glu taken up by astrocytes is converted to glutamine (Gln) by the action of glutamine synthetase (GS), then Gln is released to the extracellular space to be taken up by excitatory pre-synaptic neurons. Gln recovered by the neurons regenerates Glu under the action of phosphate-activated glutaminase (PAG) to form a Glu–Gln cycle [8, 9]. The return of the Glu precursor (glutamine) and Glu reuptake maintain the Glu level of glutamatergic pre-synaptic neurons [10]. In neurons, Glu is transported into synaptic vesicles (SVs) by vesicular glutamate transporters (VGLUTs) that play a physiological role [11, 12]. During neurotransmission, SVs are recovered by endocytosis and refilled with the neurotransmitter Glu for a new round of exocytosis, thereby forming a complete SVs involved in the glutamate metabolism cycle [13, 14] (Fig. 1). Synaptic vesicle cycle plays an important role in the process of glutamate metabolism. And the homeostasis of the Glu metabolism cycle is maintained by a group of transporters present in the plasma membrane on SVs [15]. The release of Glu depends on the transport of this amino acid to SVs [16]. To maintain synaptic efficacy, recirculating SVs are supplemented with glutamate by VGLUTs, whereas glutamate-filled SVs are dependent on the activity of VGLUTs. Therefore, VGLUTs play an important role in neuronal glutamate delivery.

This review will start from the Glu cycle, introduce the important role of Glu transporter VGLUT1 in Glu transport and metabolism, physiological distribution and transport Glu mechanism. It mainly describes the mechanism of action of VGLUT1 expression changes in a series of central and peripheral nervous system diseases such as learning and memory disorders, Alzheimer’s disease and Parkinson’s disease et al.

## Vesicular glutamate transporters

Over the past few decades, three proteins have been identified and characterized with the ability to package Glu into presynaptic vesicles. So, VGLUTs are key molecules for the incorporation of glutamate in synaptic vesicles across the nervous system. They are VGLUT1-3 that are encoded by the solute vector gene SLC17A6-8. VGLUT1 was originally named brain-specific Na^+^-dependent inorganic phosphate co-transporter (BNPI), which is mainly expressed in brain [17]. And VGLUT2 was similar to VGLUT1 and named as Differentiation-associated Na(+)-dependent inorganic phosphate co-transporter (DNPI). In addition, VGLUT1 (SLC17A7) and VGLUT2 (SLC17A6) are expressed in glutamatergic neurons of the brain including amygdala, cerebellum, cerebral cortex, hippocampus, frontal lobe, medulla, occipital lobe, putamen and temporal lobe. However, comparing to VGLUT1, VGLUT2 also expressed in caudate nucleus, spinal cord, substantia nigra, subthalamic nucleus, and thalamus. However, unlike VGLUT1 and VGLUT2, VGLUT3 (SLC17A8) can be found not only in presynaptic terminals, but also, more rarely, postsynaptically on dendrites and in cell bodies. And It is expressed in specific neurons and use for other transmitter (such as acetylcholine and serotonin) definition [18–22]. There are many characteristics including gene name, atomic mass, number of amino acids et al. (see Table 1) Immunocytochemical localization has revealed that VGLUTs located on SVs at the end of the axon, which form a gray type I (asymmetric) synaptic contact, a putative excitatory synapse that acted in these structures [23]. In addition, the mRNA and immunoreactivity of VGLUTs are mainly distributed in a complementary manner to different excitatory neuronal populations; for example, VGLUTs is used for glutamate uptake in the excitatory axon ends of cortical cortex or intracortical fibers [24, 25]. Among them, VGLUT1 is the main isotype, with the largest proportion and the most functions, accounting for most excitatory glutamatergic terminals in the CNS.

**Table 1:** The properties and characteristics of VGLUTs

	VGLUT1	VGLUT2	VGLUT3
Gene name	SLC7A7	SLC7A6	SLC7A8
Atomic mass (kDa)	61.6	64.4	65
Number of amino acids	560	582	589
C-and N-terminal domains	Intracellular	Intracellular	Intracellular
Number of transmembrane domains	6–12	12	10
Expression organ sites	Brain	Brain	Small intestine/brain/colon
Highest expression organ(s)	Anterior cingulate cortex	Lateral nuclear group of thalamus	Small intestine Peyer’s patch
Functions	Mediates the uptake of glutamate into synaptic vesicles at presynaptic nerve terminals of excitatory neural cells. May also mediate the transport of inorganic phosphate		

## Physiological functions of VGLUT1

**Fig. 2:**
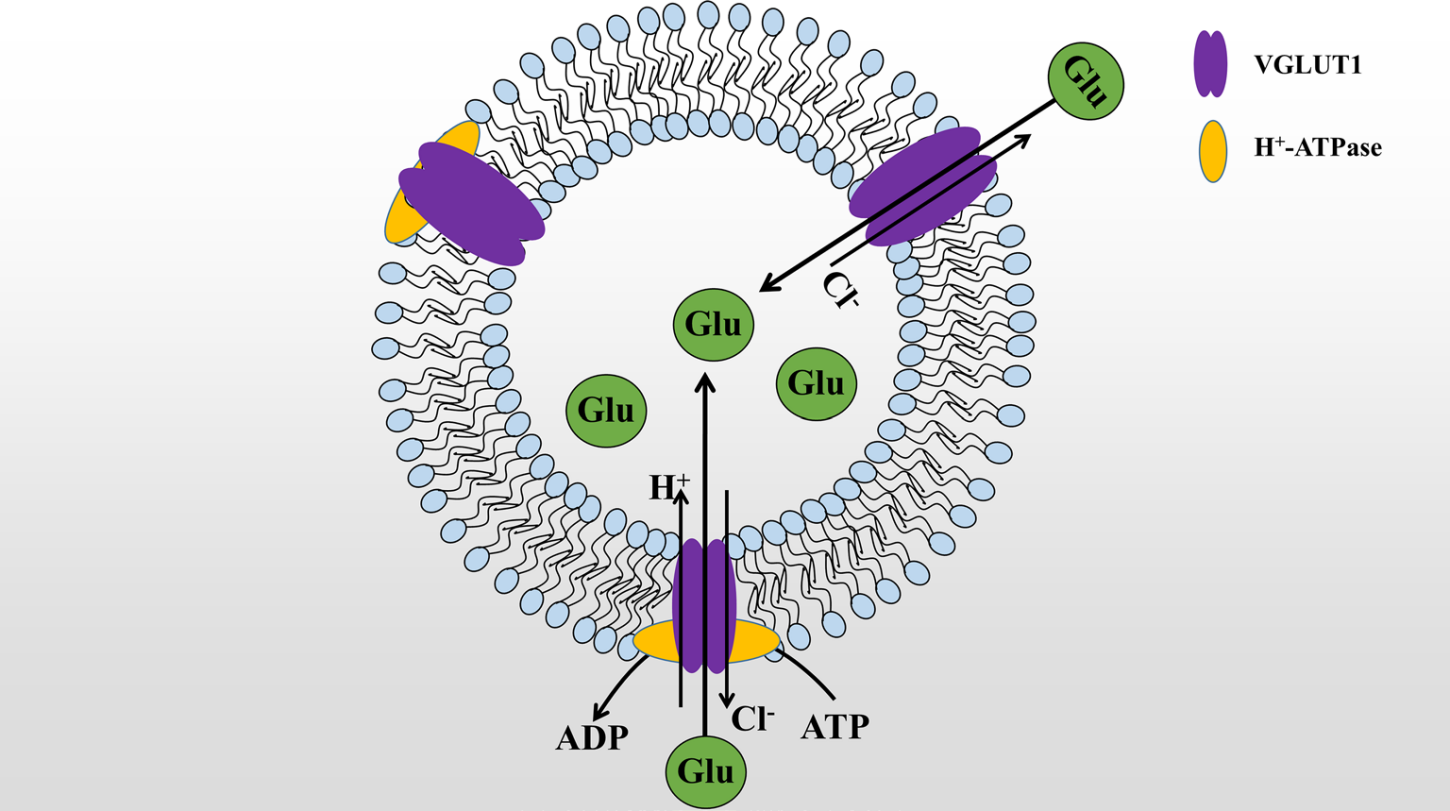
VGLUT1 transport glutamate (Glu) mechanism diagram. VGLUT1 use proton electrochemical gradient generated by vascular ATPase to carry the glutamata anion (Glu) into the interior of synaptic vesicles. The outflow of chloride ions in synaptic vesicles increases the transport efficiency of VGLUT1. Another explanation is that VGLUT1 transports chloride ions out and transfers equal amounts of Glu. In summary, VGLUT1 acts as a transporter through two pathways

The VGLUT1 is preferentially associated with the membranes of synaptic vesicles and functions in glutamate transport. However, how does VGLUT1 transport glutamate still be unclear? So some studies have shown by live-cell imaging with pH and chloride-sensitive fluorescent probes in cultured hippocampal neurons of wild-type and VGLUT1-deficient mice that in SVs VGLUT1 functions as a Glu/proton exchanger associated with a channel-like chloride conductance. VGLUT1 exerts a transport function by hydrolyzing a proton gradient generated by adenosine triphosphate (ATP) with a V-type H^+^-ATPase, and H^+^ flows into the synaptic vesicles by ATPase hydrolysis, thereby enhancing the acidity in the membrane to form a pH gradient; The influx of protons causes the membranous membrane to produce a corresponding change in membrane potential, providing power to transport Glu [26–29]. In addition, Cl^−^ and pH-sensitive fluorescence sensors demonstrated VGLUT1 as a transporter in SVs as a Glu/H^+^ exchanger associated with stoichiometric uncoupled Cl^−^ conductivity [30]. Their results revealed the transport mechanism of VGLUT1 under physiological conditions and provide a framework for how to regulate synaptic strength by regulating Glu transport through Cl^−^ and pH. Martineau et al. [31]. found a channel-like Cl-conductance in VGLUTs to further verify the above studies. It can be explained that the final filling level of SVs can be controlled by adjusting the Cl^−^ flux, i.e. the amount of Cl^−^ efflux is exchanged for Glu (Fig. 2).

## The effect of VGLUT1 in central nervous system diseases

In recent years, the number of patient suffering neurological diseases is increasing. Most of them have not been elucidated due to the complexity of their pathogenesis. And there are no specific treatments in clinical practice to them. Therefore, these diseases are increasingly threatening human health. Under normal conditions, Glu plays an important role in synaptic plasticity, learning and memory, and it plays a key role in the pathophysiology of neurological diseases under pathological conditions. VGLUT1, one of the Glu transporters, is responsible for transporting Glu to SVs, regulating the amount of Glu released into the synaptic cleft. Moreover, the expression level of VGLUT1 determines the amount of Glu that is filled into the vesicles and released to regulate neurotransmission. Therefore, VGLUT1 plays an important role in the central nervous system. And it can affect the development of a variety of neurological diseases [32–34]. However, there is increasing evidence that the glutamatergic system is at the heart of the neurobiology and treatment of these diseases. It is known to be a potent neuronal excitoxin that triggers rapid or delayed neurotoxicity. VGLUT1 is a specific biochemical marker of glutamatergic neurons and glutamatergic synapses. Therefore, the study of VGLUT1 can reveal the pathogenesis and preventive measures of many neurological diseases and propose new ideas [35–37]. Next, we will elaborate on the role of VGLUT1 in learning and memory disorders, Alzheimer’s disease, Parkinson’s disease and other central nervous system diseases.

### The action of VGLUT1 in learning and memory disorders

The hippocampus and cerebral cortex are brain regions that are critical to learning and memory, and VGLUT1 is also distributed in these two regions [38, 39]. There is a large body of literature indicating that VGLUT1 plays a key role in the learning and memory of the central nervous system [40]. VGLUT1 participates in learning and memory mainly by affecting synaptic Glu transport and long-term potentiation.

Preclinical and clinical studies have linked changes in Glu neurotransmission to cognitive impairment, which may be related to presynaptic changes in VGLUT1-dependent Glu synaptic transmission [41, 42]. The expression pattern of VGLUT1 determines the level of excitatory synaptic vesicle filling (i.e., glutamate quantal size) and directly affects Glu receptor and glutamatergic synaptic transmission. Genetic inactivation of VGLUT1 significantly reduces glutamatergic neurotransmission in cortical and hippocampal neurons, and a specific reduction in Glu release observed in cultured hippocampal neurons of VGLUT1^−/−^ mice [19]; At the same time, overexpression of VGLUT1 increased presynaptic Glu release beyond the wild-type (WT) value [43, 44]. Memory impairment exhibited by VGLUT1^−/−^ mice is associated with down-regulation of VGLUT1-dependent glutamatergic transmission in the cortical brain region of VGLUT1 as the major isoform. This leads to decrease in the accumulation efficiency of Glu in SVs, and a decrease in synaptic availability of Glu during neurotransmission and participation in learning and memory dysfunction. Linear correlation analysis showed that VGLUT1 expression was positively correlated with learning and memory ability [40, 45].

VGLUT1 also participates in learning memory by affecting long-term potentiation (LTP). VGLUT1^−/−^ mice show reduced flexibility when re-learning the new platform position in the water maze. Further studies have shown that in new object recognition tests, VGLUT1^−/−^ mice show normal short-term memory but normal long-term memory impairment [45]. Some researchers have reported that decreased VGLUT1 in VGLUT1^−/−^ knockdown mice caused a decrease in LTP and spatial reversal learning. It was further confirmed that deletion of VGLUT1 results in impaired hippocampal LTP in the CA1 region in vitro [46]. Reducing the expression of VGLUT1 in the hippocampus can lead to changes in dendritic structure, which means that synaptic connectivity reduces neurofibrillary lesions leading to LTP in adult mice, causing spatial learning and memory impairment [47]. However, the decrease in LTP amplitude in VGLUT1^−/−^ mice is most likely a result of reduced Glu release during high frequency stimulation due to lower fill levels of individual vesicles or poor vesicle recycling.

### The action of VGLUT1 in Alzheimer’s disease

According to epidemiological analysis, more than 40 million people worldwide suffer from Alzheimer’s disease (AD) and it will become the leading cause of death [48, 49]. The pathological features of AD are plaque, tangles, cell and synaptic loss, and the downstream consequences of these pathological processes include neurodegeneration with loss of synapses and neurons leading to visible atrophy, the damage of glutamatergic system in these pathological mechanisms is one of the reasons that cannot be ignored [50]. The clinical dementia score (CDR) was used to assess that VGLUT1 reduction was highly correlated with cognitive impairment. These findings suggest that the glutamatergic system is severely damaged in the cerebral cortex of AD patients, and that this injury is closely related to the progression of cognitive decline [51, 52]. The prefrontal cortex is deeply related to memory and cognition. And play a normal physiological role through a wide range of internal and subcortical regions of convergence input [53, 54]. And the concentration of VGLUT1 in the prefrontal cortex can be used as an early marker of cognitive decline. Synaptic loss is considered a hallmark of AD, and VGLUT1, as one of the synaptic markers in the prefrontal cortex of AD patients, plays a key role in synaptic loss [55]. Studies have compared the ratio of VGLUT1/synaptophysin in the prefrontal cortex of the control and AD patients, suggesting that the results reflect loss of synaptic protein rather than loss of nerve endings, and surviving synaptic terminals express lower levels of VGLUT1. Therefore, a large loss of VGLUT1 in the prefrontal cortex of AD patients may significantly reduce the intensity of glutamatergic transmission, thereby cause devastating consequences for cognitive function by disconnecting this region from other supplied cortical and subcortical regions [56, 57]. AD begins with a synaptic deficit. This pathological process also exists in the hippocampus. The adenosine A2A receptor (A2AR) in the hippocampus is mainly located in synapses that control synaptic plasticity. After treatment of the hippocampus of animals with a selective A2AR antagonist, the decrease in synaptoxin (Syntaxin-I) and glutamatergic synaptic marker VGLUT1 was observed. In addition, the number of synaptophysin-positive hippocampal synaptosomes that were also immunopositive for VGLUT1 was reduced, indicating a specific change in the glutamatergic end in the early model of AD [58].

At the same time, significant reductions in synaptophysin and VGLUT1 were found in the parietal and occipital cortex of the AD model, and these changes were associated with cortical entanglement [59]. This phenomenon occurs in a region that is rarely affected by AD pathology, represented by atrophy and cell loss. According to this, VGLUT1 plays a important role in the pathological process of AD.

In addition, AD is associated with the degradation of central cholinergic and Glu transport, which is associated with progressive memory loss and accumulation of amyloid beta (Aβ). Aged garlic extract (AGE) can alleviate the impairment of working memory by modifying Aβ-induced cholinergic neuron VGLUT1 in rat hippocampus [60, 61]. Bell et al. [62] reported the presence of large dystrophic globular VGLUT1 positive ends near the amyloid plaque in the frontal of AD. At the same time, studies have reported that synaptophysin is mainly associated with VGLUT1 positive ends, further demonstrating that VGLUT1 is involved in the formation of amyloid plaques to accelerate the progression of AD [63, 64].

However, the improvement of people’s living standards has led to the development of some metabolic diseases and cardiovascular diseases. Rodriguezperdigon et al. [65] proposed that AD is associated with certain metabolic disease factors and that the progression of the disease is exacerbated by abnormal regulation of VGLUT1. Furthermore, studies have reported that activation of JNK (c-Jun N-terminal kinase) in AD patients inhibits insulin signaling, which results in decreased expression of VGLUT1, thus leading to Glu deficiency in AD. Modulation of VGLUT1 activity by regulation of pJNK can be considered as a potential therapeutic target for the treatment of metabolic disorders in AD. Juge et al. [66]. found that excessive levels of ketones regulate VGLUT1 activity, suggesting that defects in insulin signaling can convert neuronal metabolism to produce ketone bodies, which in turn may result in decreased expression of VGLUT1, resulting in reduced Glu release. This leads to a lack of Glu in AD and accelerates the progression of the disease. At the same time, Hascup ER and other studies found that high-fat diet (HFD) induces changes in central insulin signaling that can be metabolized to produce ketone bodies, which in turn may cause a decrease in VGLUT1 expression in the hippocampus, resulting in a decrease in released Glu. Further demonstration of the Glu deficiency described in AD [67, 68].

Kalaria et al. [69] have demonstrated that cerebral ischemia is the main cause of late development of AD. And in the AD rat model of cerebral ischemia, Khan et al. [70] reported a decrease in VGLUT1 protein level in the hippocampal CA1 region 7 days after ischemic injury in rats. Thus, damage to the glutamatergic system exacerbates the progression of AD.

### The role of VGLUT1 in Parkinson’s disease

Parkinson’s disease (PD) is characterized by progressive degeneration of the substantia nigra pars compacta (SNc) neurons and is associated with abnormal glutamatergic activity [71]. In PD, progressive degeneration of DA-capable cells of SNc leads to an imbalance within the cortical-basal ganglion loop and is associated with abnormal glutamatergic innervation in the brain [72, 73]. El Arfani et al. [74], indicating changes in different transport molecules of extracellular glutamate levels in bilateral SNc in clinically relevant rat models. In fact, bilateral SNc lesions reduced VGLUT1 expression and significant changes occurred 2 weeks after injury. Kashani’s team [75] reported that the reduction of VGLUT1 levels in the prefrontal and temporal cortex of PD patients was consistent with animal model studies and also demonstrated the enormous role of VGLUT1 in glutamatergic damage in PD.

Furthermore, in a mouse model of Parkinson’s disease, the expression of VGLUT1 increases with the loss of dopamine; However, a significant decrease in 1-methyl-4-phenyl-1,2,3,6-tetrahydropyridine (MPTP) induction and an increase in VGLUT1 in the dorsolateral (DL) striatum. However, the decrease in behavior after PD treatment with MPTP may be related to changes in cortical–cortical Glu function and striatum Glu VGLUT1 input. This shows that VGLUT1 plays a major role in the pathological process of PD [76]. Previous studies have shown that electroacupuncture (EA), as one of the treatments for PD, can promote the recovery of PD motor function [77]. Clinically, the subthalamic nucleus (STN) is a key target for deep brain stimulation in the treatment of PD, and VGLUT1 plays an important role in the regulation of glutamate in cortical STN [78]. EA treatment reversed the down-regulation of VGLUT1 in STN induced by 6-hydroxydopamine (6-OHDA) [79]. EA can alleviate motor symptoms and up-regulate VGLUT1 in the ipsilateral STN of rats with PD, suggesting that up-regulation of VGLUT1 in STN may be related to the effect of EA on the motor symptoms of Parkinson’s disease through the cortico-STN pathway [80].

PD is characterized by interference with glutamatergic neurotransmission in the striatum. Two-way and bilateral changes in the expression levels of two VGLUT1 proteins in the striatum of the PD mouse model indicate a different and time-dependent change in glutamatergic transmission of striatal afferent nerves between the two types of changes [80]. Studies in rats have shown that the glutamatergic cortico-striatum pathway is VGLUT1-positive, and the number of cortical-striatum perforation synapses in PD patients is increased by 88%. Increases in perforated synapses may be associated with increased concentrations of VGLUT1 at each end [20–22, 75, 81]. The glutamatergic pathway plays a key role in the functional organization of the neuronal circuits involved in PD. These studies suggest that profound changes in Glu transmission in PD, which may lead to disease-related motor and cognitive disorders, should be considered in the treatment of PD. In conclusion, PD has many pathogenesis, but VGLUT1 plays an important role in these mechanisms. Changes in protein expression of VGLUT1 also contribute to the progression of PD.

### The action of VGLUT1 in other central nervous system diseases

Many studies have shown that VGLUT1 plays an important role not only in the mechanism of action of common central nervous system diseases such as learning memory disorders, AD and PD, but also in other central nervous system diseases, including depression, Schizophrenia and so on.

Studies in VGLUT1 heterozygous mice have shown that short-term chronic mild stress and a decrease in VGLUT1 in VGLUT1^−/−^ mice affect Glu transmission and induce depression-like behavior and impaired memory recognition [82, 83]. Many studies have linked depression to an increase in the rate of excitatory inhibition in the forebrain, suggesting that a decrease in VGLUT1 levels in the forebrain affects the Glu/GABA cycle and leads to helpless behavior. The reduction of VGLUT1 in this cycle is a potential factor in enhancing the depressive phenotype in animal models [84–86]. Tordera et al. [45] studied the possible effects of VGLUT1 transporter down-regulation on anxiety, depression-like behavior and learning, and proposed VGLUT1-mediated presynaptic changes in glutamatergic synapses. In certain areas of the brain it will lead to behavioral manifestations similar to certain aspects of mental and cognitive impairment. The pleasure and helpless behavior exhibited by depressed mouse models may be associated with decreased Glu delivery in those regions where VGLUT1 is the major isoform [87]. Consistent with this, some autopsy studies have shown a reduction of cortical VGLUT1 in depressed subjects and the clinical manifestations of excitatory inhibitory imbalance in the cortex of patients with depression [84, 88, 89].

In addition, post-mortem studies using various methods and targeting several molecular targets provide increasing evidence that glutamatergic neurotransmission is affected in schizophrenia. In patients with schizophrenia, especially in elderly subjects, the presynaptic glutamatergic marker VGLUT1 is reduced [90]. In schizophrenia, a decrease in VGLUT1 mRNA expression in the entorhinal cortex (ERC) suggests that loss of presynaptic innervation may represent a loss of association between these regions, leading to changes in mood regulation. The researchers also found a decrease in VGLUT1 mRNA in the hippocampus, suggesting a decrease in the activity or number of glutamatergic synapses [91]. It can be seen that the change of VGLUT1 in schizophrenia also damages the normal glutamatergic system including synaptic activity, the amount of Glu transport, etc. and accelerates the development of the disease.

## The effect of VGLUT1 in peripheral nervous system

Several decades of research established that Glu is not only the major excitatory neurotransmitter in the mammalian central nervous system (CNS) [92] but also the peripheral nervous system (PNS), including dorsal root ganglion (DRG), Trigeminal ganglion (TG) and spinal cord neurons [93, 94]. VGLUT1 fill neurotransmitter vesicles with glutamate and it is shipped to peripheral terminals. However, VGLUT1 is immunohistochemically detected in peripheral nerve endings, involving sensory receptors transducing either nociceptive, thermal or mechanical stimuli and it intimates Glu release from peripheral primary afferent terminals. The released glutamate, putatively from the peripheral nerve endings, may contribute to sensitized nociception or pain pathology [95].

Some researches prove that VGLUT1’s preference for medium-, especially large-sized DRG neurons indicates its functional implication in low-threshold mechanoreception [96]. Large DRG neurons serve mechanoreception and they send VGLUT1-IR, myelinated primary afferents to terminate in spinal cord [97]. Liu et al. [98] reported that VGLUT1 synapses are involved in pre-motor neuron-mediated responses in the spinal cord, so this study directly certifies the VGLUT1’s involvement in mechanoreceptive sensation. TG resembles DRG in neuronal VGLUTs’ profile, with nearly all somata expressing VGLUT1. However, VGLUT1 may also engage active nociceptive TG neurons. Because of VGLUT1 was induced by lipopolysaccharides and co-expressed with ionotropic purinergic receptor family (P2X) member (s) in small-sized TG neurons [99]. According to some studies proves that VGLUT1 and VGLUT2 complementary existence in PNS to works on nociception. Thus, it is reasonable to presume that the peripheral VGLUT1 and VGLUT2 are more implicated in proprioception and nociception, respectively. Centrally, the two VGLUTs isoforms are also segregated in neuronal populations, probably used for coding of distinct sensory signals and changes in VGLUT1 expression will affect the complementary role of the two. In addition, VGLUT1 is remarkably expressed in muscle spindle afferent endings, and it is involved in skeletal muscle proprioception [100]. Only VGLUT1-IR was detected in intrafusal fibers of masseteric spindles. So VGLUT1 is the solo functionally engaged in muscle proprioception [101]. A full understanding of VGLUTs’ function in transmission and processing of neural signals from different modalities necessitates an all-around knowledge on these transporters. Finally, future endeavor is required to develop new drugs targeting VGLUT1 to relieve pain, since intervention of glutamate transmission clearly affects pain sensation.

## Conclusions

In this review, it summarizes the VGLUT1, one of the important Glu transporters, from the maintenance of the steady state of glutamatergic system. And it fully demonstrates the important role of VGLUT1 in pathogenesis, disease progression and prophylaxis. In the above-mentioned central nervous system diseases, damage of the glutamatergic nervous system is one of the main causes of disease and accelerated disease progression. The important transporter VGLUT1 plays an important role in this pathological process, and this is closely related to its distribution and physiological function as well as the mechanism of Glu transport. The disruption of the transport mechanism of VGLUT1 directly affects the transport efficiency and transport amount of Glu, causing the destruction or disorder of the glutamatergic system, which ultimately leads to the development of the above diseases. Although the research on VGLUT1 has been deepened, however, with the environmental pollution and aging population increased has accelerated the occurrence and development of similar central nervous system diseases. But, at present, there are few studies on the impact of VGLUT1 on environmental pollution or aging population.

In addition, the research on the mechanism of VGLUT1 in synaptic plasticity, pathological changes of neuronal synapses, transport velocity of synaptic vesicles and filling size is still insufficient. From above all, further researches and argumentations are needed. The effect of VGLUT1 on the EA treatment of PD may promote the recovery of motor function, however the mechanism is not fully understood. And whether changing the expression of VGLUT1 in the PD model affects the change of Glu content in STN has not been further studied. So, these need to be researched in the future and applied to the majority of patients to contribute to their treatment. However, there are no studies on epigenetic aspects including VGLUT1 mRNA methylation, protein modification, etc. Just started research on gene knockouts, at the same time, it only shows the change of the glutamatergic system in the case of VGLUT1 knockout. There is no specific proof of which genetic mode of action, whether through transcriptional level, or through translational level, or a series of studies in changes of protein modification are still zero, but also need further research and solutions.

Promoting neuroprotection by regulating the function of VGLUT1 may be an effective intervention and treatment strategy and develop specific drugs for this and related mechanisms. It provides a new approach to the treatment of central nervous system diseases associated with Glu transport. This will bring new dawn to the Glu-related central nervous system diseases, and provide an updated therapeutic target for shortening the pathogenesis of the above-mentioned similar diseases and slowing the progression of the disease.

## Data Availability

Not applicable.

## Abbreviations

AGE: Aged garlic extract
AD: Alzheimer’s disease
BNPI: Brain-specific Na^+^-dependent inorganic phosphate cotransporter
CNS: Central nervous system
DRG: Dorsal root ganglion
EA: Electric acupuncture
Gln: Glutamine
Glu: Glutamate
GS: Glutamine synthetase
LTP: Long-term potentiation
PAG: Phosphate-activated glutaminase
PD: Parkinson’s disease
PNS: Peripheral nervous system
TG: Trigeminal ganglion
VGLUTs: Vesicle glutamate transporters
VGLUT1: Type 1 vesicle glutamate transporter
SV: Synaptic vesicle
